# Selective PPARδ Agonist GW501516 Protects Against LPS-Induced Macrophage Inflammation and Acute Liver Failure in Mice via Suppressing Inflammatory Mediators

**DOI:** 10.3390/molecules29215189

**Published:** 2024-11-02

**Authors:** Hyun-Joung Lim, Hyun Jeong Kwak

**Affiliations:** 1 Division of Cardiovascular Diseases Research, Department of Chronic Diseases Convergence, National Institute of Health, Cheongju 28159, Republic of Korea; hjlim1121@korea.kr; 2 Department of Bio and Fermentation Convergence Technology, Kookmin University, Seoul 02707, Republic of Korea

**Keywords:** peroxisome proliferator-activated receptor δ, GW501516, acute liver failure, extracellular-signal-regulated kinase1

## Abstract

Inflammation is critical in the development of acute liver failure (ALF). Peroxisome proliferator-activated receptor delta (PPARδ) regulates anti-inflammatory responses and is protective in several diseases such as obesity and cancer. However, the beneficial effects and underlying mechanisms of PPARδ agonist GW501516 in ALF remain unclear. This study investigated the molecular mechanisms underlying the anti-inflammatory effects of GW501516 in macrophages and assessed its protective potential against lipopolysaccharide (LPS)/galactosamine (GalN)-induced ALF. In vivo administration of GW501516 significantly reduced LPS/GalN-induced hepatotoxicity, as evidenced by lower mortality, decreased liver damage, and attenuated secretion of IL-1β, IL-6, and TNF-α. GW501516 treatment also decreased LPS-induced nitric oxide synthase 2 (NOS2) expression and nitric oxide (NO) production in RAW264.7 cells, an effect reversed by PPARδ siRNA. Additionally, GW501516 inhibited LPS-induced phosphorylation of p38 and c-Jun N-terminal kinase (JNK), suggesting that inactivation of these MAPKs contributes to its effects. The secretion of IL-6, TNF-α, and NF-κB DNA-binding activity were also suppressed by GW501516, while the nuclear translocation of the NF-κB p65 subunit was unaffected. In conclusion, our findings suggest that GW501516 exerts protective effects in ALF by inhibiting the production of inflammatory mediators. Therefore, GW501516 may act as a potential agent for developing anti-inflammatory therapies for ALF.

## 1. Introduction

The peroxisomeproliferator-activated receptors (PPARs) family consists of three structurally similar isoforms, namely α, δ(β), and γ [1]. Among these isoforms, PPARδ is known to be ubiquitously expressed in various tissues [2] and has been implicated in a wide range of biological processes, including, but not limited to, lipid metabolism [3], placental development [4], skin wound healing [5], insulin resistance [6], and neointimal hyperplasia [7]. Another area where the effect of PPARδ has been studied quite extensively is the inflammatory response. The anti-inflammatory effect of PPARδ has been reported in a variety of cells, including endothelial cells [8,9], cardiomyocytes [10], and, the key cells modulating inflammatory process, macrophages [11]. Specifically in macrophages, it has been reported that the activation of PPARδ decreases nuclear factor kappa-light-chain-enhancer of activated B cells (NF-kB) activity and initiates dissociation of B-cell lymphoma 6 protein (BCL-6) from PPARδ, leading to the transcriptional suppression of pro-inflammatory factors such as monocyte chemotactic protein-1 (MCP-1), MCP-3, and interleukin 1 beta (IL-1β) [12]. Furthermore, the ablation of PPARδ inhibited the alternative activation of macrophages, suggesting that PPARδ also plays an important role in the anti-inflammatory M2 transition of macrophages [13].

Several PPARδ agonists, including GW0742 and MBX-8025, have shown anti-inflammatory and metabolic effects in diverse diseases, such as metabolic disorders, cardiovascular disease, and brain disease [14,15,16]. Notably, these agonists have shown potential in ameliorating hepatic inflammation and fibrosis, indicating their therapeutic promise in liver diseases, including non-alcoholic fatty liver disease (NAFLD) [17]. However, their specific roles in acute liver failure (ALF) have not been reported. GW501516 is a potent and selective PPARδ agonist known for its ability to modulate lipid metabolism, enhance mitochondrial biogenesis, and reduce systemic inflammation [18,19]. Originally developed to treat metabolic disorders such as dyslipidemia and obesity, GW501516 has demonstrated promise in improving insulin sensitivity and increasing fatty acid oxidation in skeletal muscle [20]. Moreover, preclinical studies suggest it may reduce hepatic steatosis and improve liver function. Nonetheless, its molecular mechanisms and specific impacts on ALF require further investigation.

ALF is a clinical syndrome represented by vasodilatation, massive hepatocyte death that can lead to multiple organ dysfunction (MOD) and death, for which liver transplantation is the only effective treatment [21]. Systemic inflammatory response (SIR) with increased production of inflammatory cytokines and immune cell mobilization are known to be important factors in determining the outcome of ALF [22]. Infections in ALF patients are common, and according to a previous study, more than half of patients with ALF displayed multiple systemic inflammatory response syndrome (SIRS) components such as abnormal body temperature, white blood cell count, and heart rate. Furthermore, the increasing number of SIRS components observed was associated with increased mortality. In this regard, immune cells, particularly monocytes and macrophages, have been identified as key players in the development and progression of ALF [23]. A recent study using a rat cecal ligation and puncture model of sepsis reported the anti-inflammatory effect of PPARδ via suppression of NF-kB signaling [24], and this might indicate that PPARδ is also effective in ALF, as it was effective in sepsis, and ALF and sepsis share some phenotypic similarities, including SIR [25]. However, the role of PPARδ in ALF and the molecular mechanisms underlying the anti-inflammatory effects of GW501516 have not been reported.

In this study, we aimed to explore the anti-inflammatory effect of PPARδ, the selective PPARδ agonist GW501516 on the regulation of inducible nitric oxide synthase (NOS2), tumor necrosis factor alpha (TNF-α), and interleukin 6 (IL-6) in lipopolysaccharide (LPS)-activated macrophages and underlying mechanisms responsible for the protective effects of GW501516. We also extended the in vivo study to investigate the effect of GW501516 on the D-GalN/LPS-induced ALF model. 

## 2. Results

### 2.1. GW501516 Reduced the Mortality and Liver Damage on LPS/D-GalN-Induced ALF in Mice

**Figure 1:**
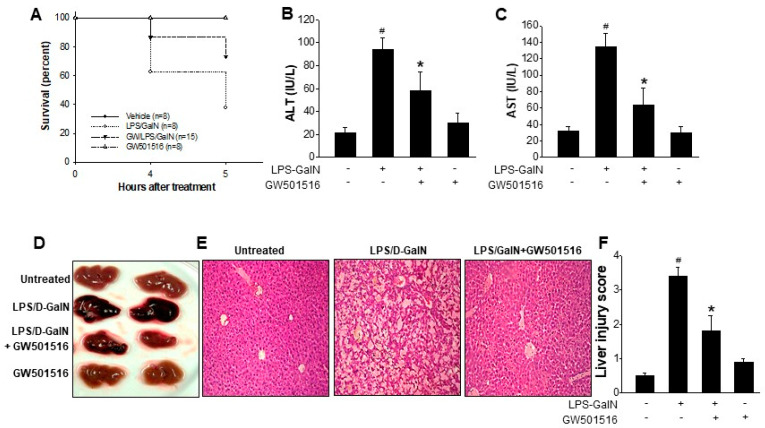
In vivo effects of GW501516 in mice with ALF induced by LPS/D-GalN. Mice were pretreated with GW501516 (2 mg/kg, ip) at 6 h prior to LPS/D-GalN (20 mg/700 mg/kg) challenge. Survival rate of mice were monitored for 5 h after LPS/D-GalN challenge (**A**). Serum levels of ALT and AST were measured (**B**,**C**). Representative gross liver morphology (**D**), representative images of H&E-stained liver section (**E**), and liver injury scores were shown (**F**). Data shown are means ± SD at least 10 different animals per group. ^#^ *p* < 0.05 versus normal mice; * *p* < 0.05 vs. LPS-GalN-treated mice.

In this study, we evaluated the effect of GW501516 on liver injury using the established LPS/D-GalN model of acute liver failure (ALF), which closely mimics clinical ALF. Mice were administered a high dose of LPS/D-GalN (20 mg/700 mg/kg), which induced ALF and resulted in a survival rate of only 37.5% within 5 h. However, pretreatment with GW501516 (2 mg/kg, i.p., 6 h before induction) improved survival to 73.3%. No mortality was observed in the vehicle (0.1% DMSO) or GW501516-treated groups (Figure 1A). Furthermore, GW501516 significantly reduced serum ALT and AST levels elevated by LPS/D-GalN (Figure 1B,C). Gross observation indicated that the livers of LPS/D-GalN-treated mice exhibited increased redness, indicating injury, whereas the livers of GW501516-pretreated mice showed reduced discoloration (Figure 1D). Histological analysis via H & E staining confirmed extensive hepatocyte necrosis and architectural disruption in the LPS/D-GalN group, both of which were markedly ameliorated by GW501516 pretreatment (Figure 1E,F).

### 2.2. GW501516 Decreased Pro-Inflammatory Cytokines and NOS2 in LPS/D-GalN-Induced ALF in Mice

**Figure 2:**
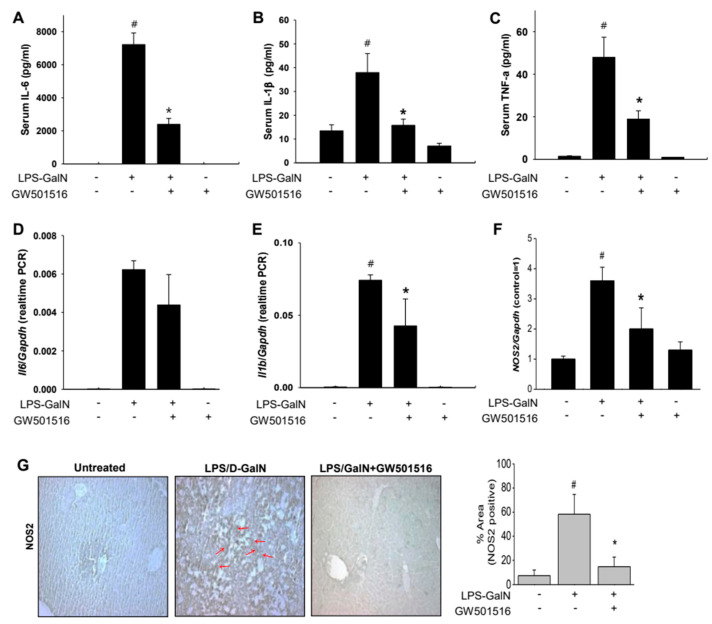
Effects of GW501516 on pro-inflammatory cytokines production in LPS/D-GalN-induced ALF mice. Mice were pretreated with GW501516 (2 mg/kg, ip) at 6 h prior to LPS/D-GalN (20 mg/700 mg/kg) challenge. Serum levels of (IL-6), IL-1β, TNF-α, (**A**–**C**) and mRNA levels of IL-6, IL-1β, and NOS2 in the hepatic tissue (**D**–**F**) were measured. Hepatic tissues performed immunohistochemistry against NOS2 antibody (red arrows). (**G**) % of NOS2-positive areas of were calculated using Image J 1.53. All values represent the mean ± SD from three or more independent experiments; ^#^ *p* < 0.05 versus normal mice; * *p* < 0.05 vs. LPS-GalN-treated mice.

Inflammatory cytokines induced by LPS/D-GalN play a critical role in liver pathogenesis. To evaluate whether GW501516 reduces inflammation following LPS/D-GalN-induced liver injury, we measured serum cytokines. Serum levels of IL-6, IL-1β, and TNF-α were significantly elevated in the LPS/D-GalN group but decreased after GW501516 pretreatment (Figure 2A–C). In liver tissue, mRNA expression levels of IL-6, IL-1β, and NOS2 were markedly increased in the LPS/D-GalN group compared to controls. GW501516 pretreatment significantly lowered IL-1β levels and tended to reduce IL-6, though not statistically significant (Figure 2D,E). Additionally, NOS2 expression, induced by LPS/D-GalN, was notably reduced following GW501516 treatment, as confirmed by both mRNA and immunohistochemistry (Figure 2F,G). These results indicate that GW501516 exerts protective effects against acute liver failure by suppressing inflammation. 

### 2.3. GW501516 Suppresses NOS2 Expression in LPS-Activated RAW264.7 Cells

**Figure 3:**
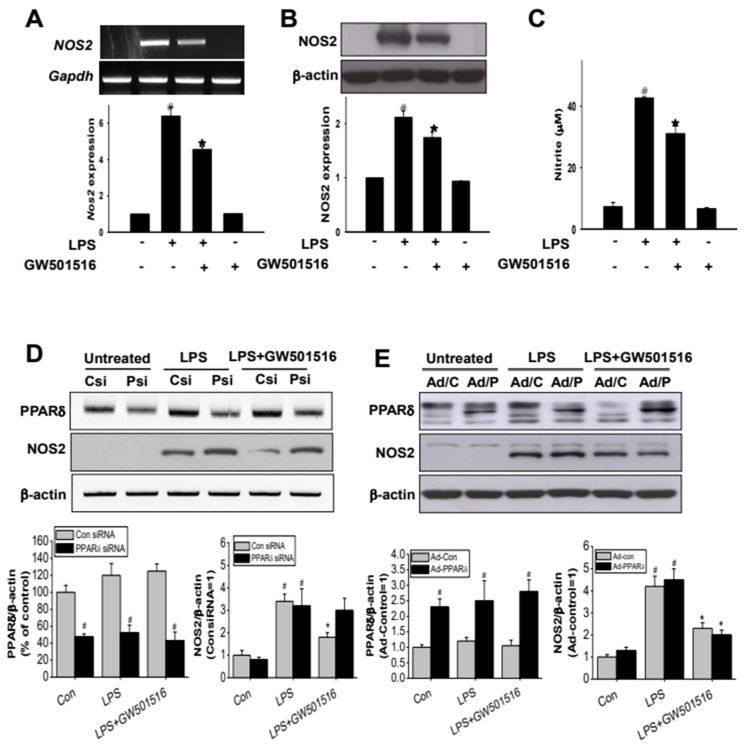
PPARδ activation decreases NOS2 expression and subsequent NO production in LPS-activated RAW264.7 cells. Relative expression levels of NOS2 mRNA (**A**) and protein (**B**); the nitrite levels (**C**) were measured in RAW264.7 cells activated with LPS (100 ng/mL), with or without pretreatment with GW501516. RAW264.7 cell transfected with PPARδ-specific siRNA were treated with LPS in the presence or absence of GW501516 pretreatment. Expression levels of PPARδ and NOS2 were detected by Western blot (**D**). Csi: scrambled control siRNA, Psi: PPARδ-specific siRNA. PPARδ-overexpressed cells were treated with LPS with or without GW501516 pretreatment. PPARδ and NOS2 expressions were detected by Western blot (**E**). Ad/C: control adenovirus, Ad/P: PPARδ adenovirus. All quantifications were analyzed using Image J. Data shown are means ± SD of at least three experiments (each performed in duplicates). ^#^ *p* < 0.05 versus normal control; * *p* < 0.05 vs. LPS-treated cells.

LPS induces inflammatory response primarily by activating Kupffer cells in the liver. Therefore, we used LPS-induced RAW264.7 cells as an in vitro surrogate model for Kupffer cells to estimate the in vitro inflammatory response. Treatment with LPS (100 ng/mL) significantly increased both mRNA and protein levels of NOS2 in RAW264.7 cells. In contrast, pretreatment with GW501516 (100 nM, 12 h prior to LPS activation) significantly, although not completely, reduced LPS-induced NOS2 expression at both mRNA and protein levels (Figure 3A,B). Additionally, the level of nitrite (NO_2_^−^), one of the major metabolites of nitrogen monoxide (NO) and an indicator of NO production [26], was greatly increased in the culture medium of LPS-activated cells, whereas treatment with GW501516 was able to abolish the induction of NO_2_^−^ (Figure 3C).

### 2.4. The Effect of GW501516 on NOS2 Regulation Is Dependent on Activating of PPARδ 

We next investigated whether the effect of GW501516 on LPS-induced NOS2 expression is mediated through activation of PPARδ. Transfection with PPARδ-specific siRNA (10 nM) reduced PPARδ expression by 52.3 ± 3.1% compared to control siRNA-transfected cells (Figure 3D lanes 1 and 2). However, LPS-induced NOS2 expression remained unaffected by PPARδ knockdown (Figure 3D, lanes 3 and 4). Furthermore, PPARδ silencing abolished GW501516′s inhibitory effect on LPS-induced NOS2 induction, while GW501516 reduced NOS2 levels in the control siRNA group (Figure 3D, lanes 5 and 6). To further elucidate PPARδ‘s role, we employed adenoviral delivery for PPARδ overexpression. As shown in Figure 3E, overexpression of PPARδ did not alter NOS2 expression in LPS-activated cells (Figure 3E, lanes 3 and 4). In contrast, GW501516 treatment of PPARδ-overexpressing cells resulted in a significant reduction in LPS-induced NOS2 expression compared to LPS treatment without GW501516 (Figure 3E, lanes 5 and 6). These findings suggest that GW501516-mediated activation of PPARδ is crucial for the regulation of NOS2 expression.

### 2.5. GW501516 Attenuates NOS2 Induction Through p38- and JNK-Dependent Mechanism 

**Figure 4:**
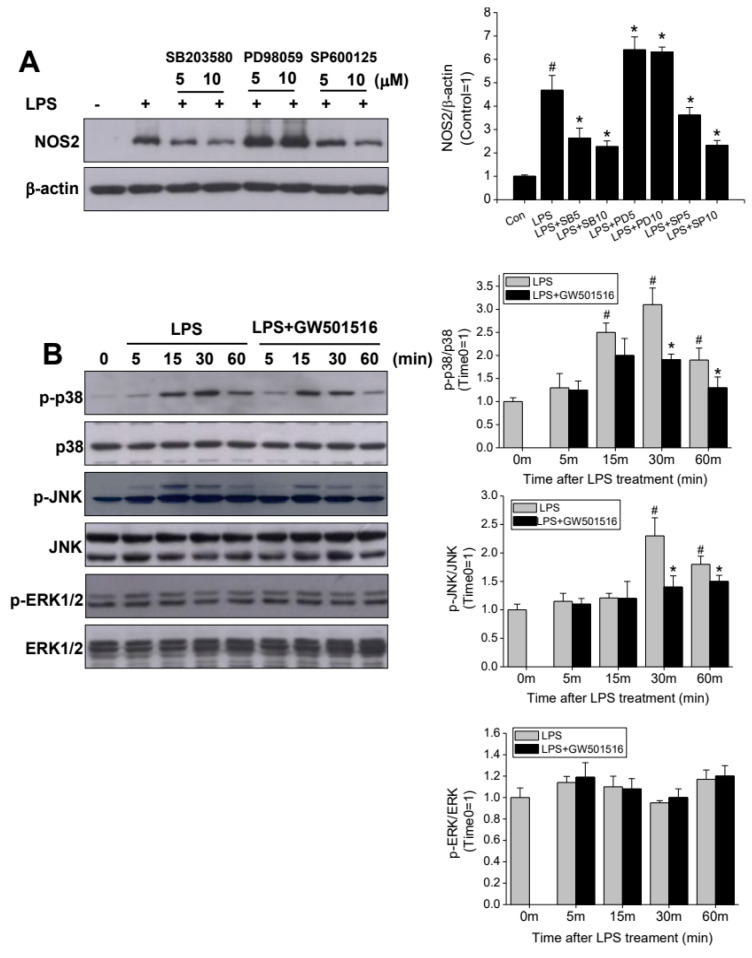
Effect of GW501516 on the regulation of MAPkinase in LPS-activated RAW264.7 cells. NOS2 expressions with p38, JNK, and ERK1/2 inhibitor treatment (**A**). Expressions of p-p38, p-JNK, and p-ERK1/2 after LPS activation with or without GW501516 pretreatment. SB203580 (**B**): p38 inhibitor, PD98059: ERK1/2 inhibitor, SP600125: JNK inhibitor. All of quantifications were analyzed by Image J1.53. Data shown are means ± SD of at least three experiments (each performed in duplicates). ^#^ *p* < 0.05 versus normal control; * *p* < 0.05 vs. LPS-treated cells.

MAPKs is a key regulatory mechanism on LPS-induced NOS2 expression. Here, to elucidate the role played by MAPKs in the inhibitory effect of GW501516, we examined JNK, p38, and ERK phosphorylation after incubation with LPS in the presence or absence of GW501516. In Figure 4A, NOS2 induction by LPS treatment was blocked by SB203580 (p38 inhibitor) and SP600125 (JNK inhibitor) treatment, respectively, but this was unaffected by an ERK inhibitor, PD98509 treatment. Next, we further confirmed whether the p38 and JNK pathways are the key mechanisms of GW501516 on LPS-induced NOS2 expression. During the activation of macrophages by LPS, both phosphorylation of p38 and JNK was increased peaking at 30 min, while no significant change was observed in the p-ERK1/2. Furthermore, both p38 and JNK phosphorylation were attenuated by pretreating with GW501516 in the presence of LPS (Figure 4B), suggesting that the p38 and JNK pathway are involved in the protective action of GW501516 on LPS-induced NOS2 induction. 

### 2.6. GW501516 Decreases TNF-α and IL-6 Production in LPS-Activated RAW264.7 Cells

**Figure 5:**
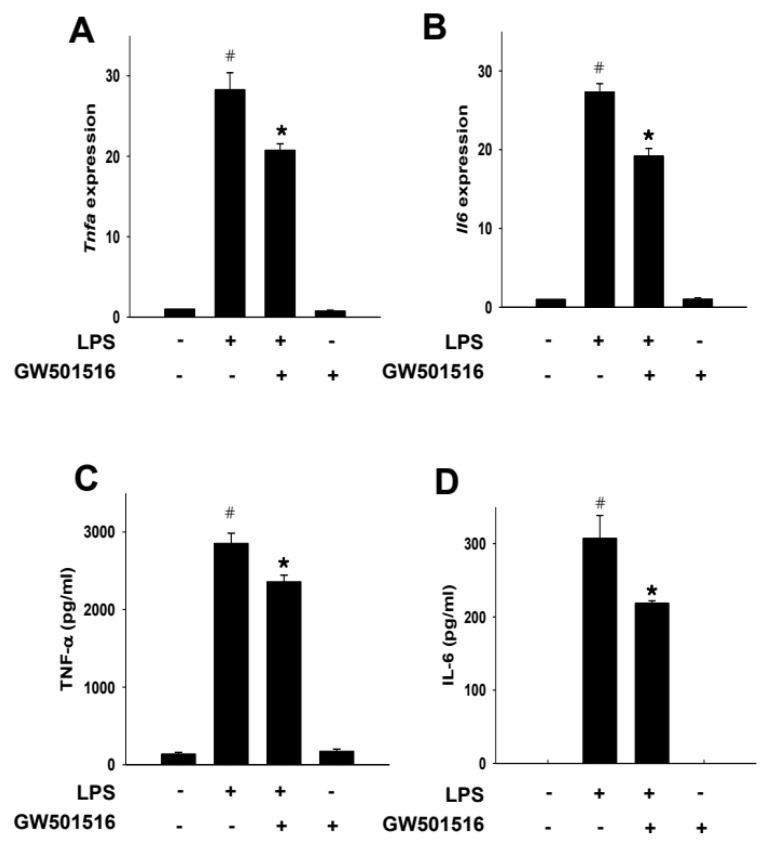
PPARδ agonist GW501516 significantly inhibits expressions of pro-inflammatory cytokines in LPS-activated RAW264.7 cells. mRNA expression of TNF-α (**A**) and IL-6 (**B**) in LPS (100 ng/mL)-stimulated cells with or without GW501516 (100 nM) pretreatment. The amount of TNF-α (**C**) and IL-6 (**D**) secreted into culture medium was measured by using ELISA kit specific for respective cytokines. All quantifications were analyzed using Image J1.53. Data shown are means ± SD of at least three experiments (each performed in duplicates). ^#^ *p* < 0.05 versus normal control; * *p* < 0.05 vs. LPS-treated cells.

We next explored the effects of GW501516 on the production of pro-inflammatory cytokines against LPS-activated macrophages. LPS treatment increased the mRNA expression of both TNF-α and IL-6, while these increases were significantly attenuated by GW501516 pretreatment (Figure 5A,B). Correspondingly, at the protein level, secretion of TNF-α and IL-6 by LPS was lessened by GW501516 pretreatment (Figure 5C,D).

### 2.7. GW501516 Exerts Its Anti-Inflammatory Effect by Inhibition of NF-κB Binding to DNA, Not the Nuclear Translocation of p65 Subunit

**Figure 6:**
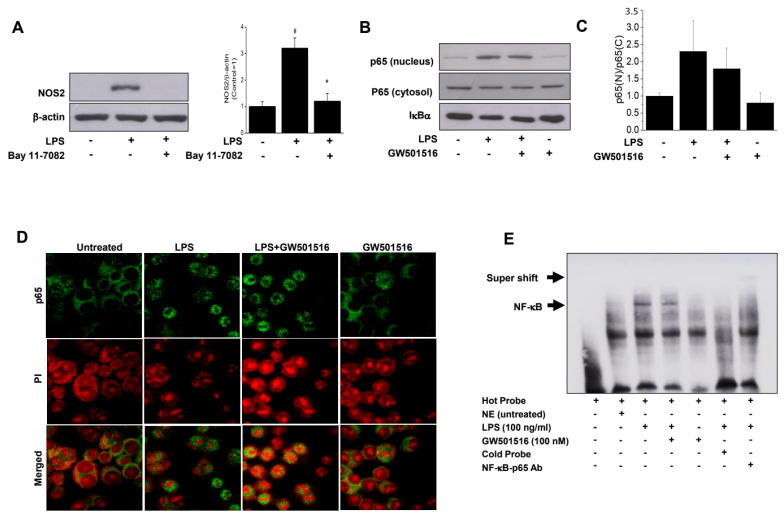
Treatment of PPARδ agonist GW501516 decreases NF-κB binding to DNA, not the nuclear translocation of p65 subunit, in LPS-stimulated RAW264.7 cells. Cells were pretreated with 3 μM BAY-117082 for 30 min and then incubated with LPS for 24 h. NOS2 expression was determined by Western blot (**A**). Cells were pretreated with GW501516 for 12 h prior to incubation of LPS for 30 min. Cytoplasmic or nuclear proteins were extracted and detected via Western blot against p65 (**B**). Quantification data of nuclear translocation of p65 subunit expressed as p65 (nucleus)/p65 (cytosol) (**C**). Representative IF-staining images of p65 subunit (**D**), and the nuclear proteins were extracted and subjected to EMSA to analyzed the p65 DNA-binding activity (**E**). Scale bar = 100 μm. Data shown are means ± SD of at least three experiments (each performed in duplicates). ^#^ *p* < 0.05 versus normal control; * *p* < 0.05 vs. LPS-treated cells.

NF-κB is a key regulator of inflammation, producing pro-inflammatory mediators such as TNF-α, IL-6, and NOS2. To evaluate the effect of GW501516 on the NF-κB signaling pathway, we first assessed the effect of Bay 11-7082 (3 μM), a well-known NF-kB inhibitor, on LPS-stimulated NOS2 induction. As shown in Figure 6A, Bay 11-7082 completely blocked NOS2 induction, confirming its NF-κB dependence. Next, we examined the inhibitory effect of GW501516 on LPS-stimulated NF-κB activation by assessing the translocation of the p65 subunit into the nucleus. LPS stimulation significantly increased nuclear p65 level, but GW501516 pretreatment did not affect this increase (Figure 6B). While GW501516 pretreatment showed a slight decrease in densitometric analysis compared to LPS stimulation, this change was not statistically significant (Figure 6C). Immunocytochemistry using an anti-p65 antibody further confirmed that GW501516 did not inhibit p65 nuclear translocation in response to LPS (Figure 6D). Given the limited effect of GW501516 on p65 translocation during LPS stimulation, we conducted EMSA for further confirmation. As shown in Figure 6E, LPS treatment enhanced the NF-κB DNA-binding activity, which was significantly reduced by GW501516 pretreatment, indicating that GW501516 inhibits NF-κB binding to DNA rather than preventing its translocation (Figure 6E).

## 3. Discussion

Activation of PPARδ has been reported to suppress LPS-induced pro-inflammatory genes such as NOS2 and TNF-α expression in macrophages [27]. Although NOS2 itself is not an effector molecule, it actively participates in the regulation of inflammatory process by producing nitric oxide (NO), which is known to mediate inflammatory process by forming reactive nitrogen species [28]. In fact, increased expression of NOS2 has been reported in diseased human liver [29,30]. In the present study, GW501516 significantly suppressed LPS-induced NOS2, as well as other pro-inflammatory cytokines such as TNF-α and IL-6, expression in line with previous reports. Such an anti-inflammatory effect of PPARδ has contributed to the suppression of NF-κB activation [6,9], which increases transcription of pro-inflammatory genes [31].

Regarding the PPARδ-mediated suppression of NF-kB, it has been reported that PPARδ agonist GW0742 inhibited IκB degradation, subsequently decreasing nuclear translocation of p65 subunit [32]. However, in our study, GW501516 decreased p65 binding to the κB element rather than decreasing nuclear translocation of p65 subunit. As for the decreased binding of p65 subunit, there can be few possible mechanisms. First, physical interaction between PPARδ and p65 subunit can be responsible. Another member of the PPAR family, PPARα, has been reported to interact with amino acid 12-317 of the p65 subunit, leading to NF-κB repression [33]. Later, although cell type is different, interaction between the PPARδ and p65 subunit also has been reported, and according to that particular study, activation of PPARδ using agonist L-165041 further increased such an interaction, preventing NF-κB-mediated expression of inflammatory genes [34]. Another possibility is modification of PPARδ by a small ubiquitin-like modifier (SUMO), so called SUMOylation. Demonstrated in the case of PPARγ-mediated suppression of LPS-induced NOS2 expression, SUMOylation of the lysine 365 (K365) of PPARγ induces its interaction with nuclear receptor co-repressor (NCoR), which subsequently hinders co-repressor complex dissociation from the promoter, maintaining a low target gene expression level [35]. A homologous sequence to the SUMOylation site of PPARγ has been identified in other PPAR family members, including PPARδ [36], suggesting that transcriptional repression of inflammatory gene by SUMOylation of PPARδ is also feasible. Nevertheless, further studies focusing on the transcriptional process are required to test such a possibility, and we are currently working on it. Furthermore, our data demonstrate that GW501516 inhibits LPS-induced NOS2 and pro-inflammatory cytokine production. However, we have not established NOS2 as the sole regulator to the observed cytokine elevation. Further research is needed to clarify the relationship between NOS2 and pro-inflammatory cytokine expression. Previous studies indicate that NO from iNOS mediates cytokine responses [37], highlighting the need for investigations into the effects of PPARδ agonists on NOS regulation and cytokine production. Limitations in our experimental design restrict our understanding; thus, future studies employing various pharmacological inhibitors targeting NOS2 are essential for elucidating its role in cytokine regulation during inflammation and for determining the molecular mechanisms of PPARδ action.

An anti-inflammatory effect of PPARδ in the liver has been reported in various disease models. For example, PPARδ-selective agonist L-165041 treatment improved steatosis and inflammation in LDLR^−/−^ mice fed Western diet [38], another PPARδ agonist GW501516 improved hepatic inflammation in a non-alcoholic steatohepatitis model [39], and yet another PPARδ agonist GW0742 reduced copper-induced liver damage [40]. Nevertheless, the effect of PPARδ in ALF, where the inflammatory response is one of the key factors in its progression and prognosis, has not been well studied. To date, many different types of chemically induced animal models simulating ALF exist [41,42]. Regarding the effect of PPARδ, the most relevant study using one of the chemically induced ALF models is the study where PPARδ showed protective effect against carbon tetrachloride (CCl_4_) induced hepatic toxicity [43], and the effect of selective PPARδ agonist GW501516 in LPS/D-GalN-induced ALF model has not been examined. 

Since the report that D-GalN treatment sensitizes animals to LPS treatment, lowering the lethal dose approximately 2500-fold [44], the combination of LPS/D-GalN has been used to induce liver damage. For the most of studies using LPS/D-GalN to induce liver damage, 10–100 μg/kg of LPS and 700–800 mg/kg of D-GalN were used [45,46,47,48,49]. However, in the present study, we used 20 and 700 mg of LPS and D-GalN, respectively, to simulate extremely harsh condition so that the effect of PPARδ agonist GW501516 in ALF can be clearly demonstrated. According to our data, animals treated with the extremely high dose of LPS started to die as soon as only 4 h after induction of liver failure with LPS/D-GalN. This was not surprising, considering a much lower dose of LPS (10 μg/kg) induced death in animals 7 h after LPS/D-GalN treatment [47,48]. 

What was significant about the survival data was that GW501516 pretreatment improved the LPS/D-GalN-induced mortality, even under such harsh conditions. In line with increased survival, GW501516 pretreatment also significantly suppressed serum pro-inflammatory cytokines such as IL-1β, IL-6, and TNF-α. Especially, the decrease in TNF-α by GW501516 is significant since TNF-α-mediated cell death is known to be the major mechanism of chemically induced, including LPS/D-GalN, liver toxicity in animals [43,48,50]. This result indicates that the potency of PPARδ activation in preventing LPS/D-GalN-induced acute liver failure might be much higher than it is currently thought to be, and it also suggests that GW501516 can be a powerful agent for managing ALF in clinical settings. Nevertheless, since only one PPARδ agonist was used in the present study, it is difficult to generally state that all the PPARδ agonists will have similar potency in preventing or improving severe ALF. One thing should be noted regarding the use of GW501516: enhanced liver fibrosis with GW501516 in animal models has been reported [51]. Without supporting data, we can only speculate at this point that prolonged use of GW501516 (daily, up to 6 weeks) might be responsible for the enhanced liver fibrosis, and it indicates that continuous and long-term use of GW501516 has a negative impact on the liver.

## 4. Materials and Methods

### 4.1. Cell Culture and Reagents

The murine macrophage cell line RAW264.7 (American Type Culture Collection, Manassas, VA, USA) cells were cultured in RPMI medium supplemented with 10% FBS. Cells were pre-treated with appropriate inhibitors 24 h prior to GW501516 treatment. Cell culture reagents and oligo(dT)_12-18_ were from Invitrogen (Carlsbad, CA, USA). LPS was purchased from Sigma-Aldrich (St. Louis, MO, USA). GW501516 was purchased from AXXORA (San Diego, CA, USA).

### 4.2. Reverse Transcriptase Polymerase Chain Reaction Analysis

The total RNA from RAW264.7 cells was extracted with an RNeasy mini kit according to the manufacturer’s recommendations (Valencia, CA, USA). From 500 ng of total RNA, cDNAs were synthesized using oligo (dT)_12–18_ primer and Accupower RT premix. Accupower RT premix and Accupower PCR premix were purchased from Bioneer (Seoul, Republic of Korea). Mouse NOS2, IL-6, TNF-α, and β-actin mRNA were amplified by PCR using the following primers: mouse NOS2; sense 5′-CAG AAG CAG AAT GTG ACC AT-3′, antisense 5′-CTT CTG GTC GAT GTC ATG AG-3′; mouse TNF-α; sense 5′-GTA GCC CAC GTC GTA GCA AA-3′, antisense 5′-AAA GTA GAC CTG CCC GGA CT-3′; mouse IL-6; sense 5′-ACA ACC ACG GCC TTC CCT AC-3′, antisense 5′-CCA CTC CTT CTG TGA CTC CA-3′; mouse GAPDH, sense 5′-GCG AGA CCC CAC TAA CAT CA-3’, antisense 5’-GAG TTG GGA TAG GGC CTC T-3’.

### 4.3. Western Blot Analysis

For Western blot analysis, LPS-induced cells with or without GW501516 pretreatment were washed with PBS and harvested. Cells were harvested in a lysis buffer containing 50 mM HEPES (pH 7.0), 250 mM NaCl, 5 mM EDTA, 0.1% Nonidet P-40, 1 mM phenylmethylsulfonyl fluoride, 0.5 mM dithiothreitol, 5 mM Na fluoride, 0.5 mM Na orthovanadate, 5 μg/mL leupeptin, and 5 μg/mL aprotinin, and incubated for 10 min at 4 °C. Protein concentrations were determined using a Bio-Rad protein assay reagent according to the manufacturer’s instructions. Whole cell lysates (10 μg of protein) were loaded into an 8 or 10% SDS-PAGE gel and then transferred to PVDF membranes (Amersham Biosciences, Piscataway, NJ, USA). Blocked membranes were then incubated with anti-NOS2 (#13120, Cell signaling technology, Danvers, MA, USA), PPARδ (#ab23673, Abcam, Cambridge, UK), p-p38 (#sc-16682, Santa Cruz, CA, USA), p38 (#9212, Cell signaling technology), p-ERK (#4370S, Cell signaling technology), ERK (#4695S, Cell signaling technology), p-JNK (#sc-6254, Santa cruz), JNK (#sc-7345, Santa cruz), p65 (#sc-372 Santa cruz) and IκBα (#ab133462, Abcam) for overnight at 4 °C. β-actin (#3700S, Cell signaling technology) was used as a loading control. After washing with TBS-T, blots was then incubated with secondary antibodies conjugated with horseradish peroxidase (HRP). Immunoreactive bands were visualized by enhanced chemiluminescence (Amersham Life Science, Buckinghamshire, UK), and band densities were quantified using UN-SCAN-IT gel ver. 5.1 (Silk Scientific, Inc., Orem, UT, USA) and normalized versus β-actin.

### 4.4. Measurement of Nitrite

Accumulation of nitrite in the medium was measured with a colorimetric assay method based on the Griess reaction (Cayman Company, Ann Arbor, MI, USA). In total, 100 μL of each sample was added to the wells. And then 50 μL of Griess reagent R1 was added, followed by an addition of 50 μL Griess reagent R2 to each of the wells. After incubating, nitrite concentration was determined by absorbance at 540 nm.

### 4.5. ELISA for IL-6 and TNF-α

An ELISA kit for detection of mouse IL-1β, IL-6, and TNF-α was purchased from R&D systems. Cells were seeded at 3 × 10^6^ cells in 100 mm culture dish and pretreated with GW501516 for 12 h in RPMI containing 10 % FBS before LPS treatment. At 24 h after LPS treatment (100 ng/mL), the culture media (50 μL) were analyzed for the presence of IL-1β, IL-6, and TNF-α according to the manufacturer’s instructions. The absorbance at 450 nm was determined using a microplate reader with SOFTmax PRO 7.5 software (Molecular Devices, Sunnyvale, CA, USA). Serum IL-1β, IL-6, and TNF-α were analyzed using a milliplex MAP kit from Millipore system.

### 4.6. Anlysis of ALT and AST 

Liver markers, including alanine aminotransferase (ALT) and aspartate aminotransferase (AST), were measured in mouse using assay kits obtained from (Abcam, Cambridge, UK) according to the manufacturer’s instructions. The absorbance (OD) were measured at 450 nm with a microplate reader, and the concentrations were determined based on the standard curve.

### 4.7. Gene Silencing with siRNA Transfection

The siRNA-targeting mouse PPARδ and control were purchased from SantaCruz (Santa Cruz, CA, USA). Using Lipofectamine RNAiMax reagent (invitrogen), 10 nM of siRNA was transfected into the RAW264.7. At 48 h after transfection, RT-|PCR was used to evaluate the silencing effect of siRNA on PPARδ.

### 4.8. Electrophoretic Mobility Shift Assay (EMSA)

Nuclear extracts were prepared and separated in 6% acrylamide native gel and then transferred to a nylon membrane (Zeta-probe GT genomic tested blotting membranes, Bio-Rad, Hercules, CA, USA). Immunopositive bands were developed and detected using Lightshift Chemiluminescent EMSA kit (Pierce, Rockford, IL, USA). Probe for the EMSA (5′-AGTTGAGGGGACTTTCCCAGGC-3′) was purchased from Bioneer (Daejourn, Republic of Korea). And the 3′ end was biotinylated using Biotin 3′ End DNA labeling kit (Pierce, Rockford, IL, USA).

### 4.9. Immunofluorescence Staining

Cultured cells were fixed with 10% formaldehyde at room temperature for 30 min and washed three times with PBS. Anti-p65 antibody (#sc-372, Santa Cruz Biotechnology, Santa Cruz, CA, USA) was used as a primary antibody in 1% BSA-PBS. The primary antibody was detected with Alexa Flour^®^ 488-conjugated goat-anti-mouse IgG (Molecular Probe, Eugene, OR, USA). PI (propidium iodide) was used as a nuclear counterstain. Immunofluorescence was visualized using a confocal laser fluorescence microscope (Carl Zeiss Inc, Thornwood, NY, USA). 

### 4.10. Immunocytochemistry

Slicing of 4 μm thickness was carried out for paraffin-embedded liver tissues, followed by deparaffinization and serialdehydration in ethanol (75%, 85%, 95%, 100%). Subsequently, antigen retrieval was made with EDTA. Sections of liver tissue were then treated at 4 °C overnight against NOS2 (1:200). Sections of liver tissue were further treated at room temperature for 30 min with a secondary antibody, followed by staining with Diaminobenzidine(DAB) and counterstaining with hematoxylin. A brownish color specified the positive areas. For microscopic examinations, a Nikon E600 upright light microscope (Melville, NY, USA) was used and the images were taken using a Nikon Coolpix 4300 digital camera. Positive area of staining quantified by Image-Pro Plus software 6.0.

### 4.11. Hematoxylin and Eosin (H&E) Staining and Histological Assessment

Liver tissues were fixed in 4% paraformaldehyde for 24 h, dehydrated with gradient ethanol, embedded in paraffin, and sectioned at 4 μm thickness. The slices were immersed in xylene I/II in sequence, then in gradient ethanol, and finally washed with water. Thereafter, the sections were stained via HE, followed by immersion in gradient ethanol and xylene I/II each, and then, the sections were observed under a light microscope. The degree of liver damage was evaluated using a histological scoring system ranging from 1 to 4, based on the extent of hepatic lobule destruction, inflammatory cell infiltration, hemorrhage, and hepatocyte necrosis. The scoring criteria are as follows: 0, none; 1, individual cell necrosis; 2, ≤30% lobular necrosis; 3, ≤60% lobular necrosis; 4, >60% lobular necrosis.

### 4.12. In Vivo ALF Model

Male C57BL6/J (8 to 9 wks old) mice, weighing 20 to 25 g, were maintained in specific pathogen-free conditions at our animal-breeding facility. LPS/GalN (20 mg/700 mg/kg) in saline was injected in a single i.p. dose into mice to induce acute liver failure. To examine whether GW501516 had anti-inflammatory effect, mice were randomized and treated with vehicle (0.1% DMSO; *n* = 8), LPS/GalN (*n* = 8), LPS/GalN plus GW501516 (2 mg/kg; *n* = 15), and GW501516 only (*n* = 8). GW501516 was administrated intraperitoneally 6 h before LPS administration. Five hours later, a blood sample was collected via orbital puncture, and the serum was prepared by centrifugation at 12,000× *g* for 20 min at 4 °C for measuring IL-1β, IL-6, TNF-α, ALT, and AST levels. The survival rate of the mice was monitored for 5 h. All the experiments were performed in accordance with the guidelines of the care and use of Laboratory Animals by National Institutes of Health in Korea (NIH-08-26). 

### 4.13. Statistical Analysis

The data are represented as means ± SEM of more than three separate experiments. Significant difference between the respective control and each experimental test condition was assessed by using Student’s *t*-test for each paired experiment. * *p* < 0.05 was considered statistically significant.

## 5. Conclusions

The PPARδ agonist GW501516 improves survival in a mouse model of acute liver failure induced by LPS/D-GalN, primarily by suppressing the expression of pro-inflammatory cytokines such as TNF-α, IL-6, and NOS2 in vivo. This anti-inflammatory effect is mediated through the inhibition of the p38 and JNK pathways and involves reducing NF-κB DNA-binding activity in vitro. These findings suggest that the efficacy of GW501516 may be significantly greater than previously understood, as its ability to enhance survival under severe conditions appears to result from the suppression of pro-inflammatory cytokine release, including TNF-α.

## Data Availability

The data presented in this study are available on request from the corresponding author.
